# Loss of a primary cilia protein ARL13B promotes TGFβ-1 induced EMT of RPE in proliferative vitreoretinopathy via increasing Smad3 expression

**DOI:** 10.3389/fcell.2025.1661658

**Published:** 2025-12-04

**Authors:** Wenjun Sui, Xinqi Ma, Yajun Gong, Kairui Qiu, Miner Yuan, Keli Mao, Xiaobing Qian, Jieting Zeng, Yujie Li, Bingsheng Lou, Xiaofeng Lin, Xiaolai Zhou

**Affiliations:** 1 State Key Laboratory of Ophthalmology, Zhongshan Ophthalmic Center, Guangdong Provincial Clinical Research Center for Ocular Diseases, Sun Yat-sen University. Guangdong Provincial Key Laboratory of Ophthalmology and Visual Science, Guangzhou, China; 2 Guangdong Basic Research Center of Excellence for Major Blinding Eye Diseases Prevention and Treatment, Guangzhou, China; 3 Shenzhen Eye Hospital, Shenzhen Eye Medical Center, Southern Medical University, Shenzhen, China; 4 Department of Ophthalmology, Sichuan Academy of Medical Sciences & Sichuan Provincial People’s Hospital, University of Electronic Science and Technology of China, Chengdu, China

**Keywords:** proliferative vitreoretinopathy, retinal pigment epithelial cells, epithelial-mesenchymal transition, ARL13B, primary cilia

## Abstract

**Background:**

Proliferative vitreoretinopathy (PVR) is a major complication of rhegmatogenous retinal detachment. Epithelial-mesenchymal transition (EMT) of retinal pigment epithelial (RPE) plays a central role in PVR pathogenesis. This study aims to investigate the effect of ADP-ribosylation factor-like 13B (ARL13B) on RPE EMT in PVR.

**Methods:**

The expression of ARL13B in PVR specimens was analyzed by immunofluorescence (IF) staining. The effect of ARL13B on RPE EMT was assessed by IF staining and Western blot. The proliferation and migration of RPE were measured with EdU and transwell and scratch assays, respectively. The EMT-related transcriptome was analyzed by bulk RNAseq. An intravitreal injection mouse model of PVR was used to investigate the role of ARL13B in PVR formation.

**Results:**

Immunofluorescence revealed significantly reduced ARL13B levels in α-SMA-positive cells as compared with Pan-CK-positive cells in an epiretinal membrane derived from retinal tears. During EMT, TGFβ1 treatment remarkably reduced ARL13B expression and shortened the length of cilia in RPE cells. In line with this, ARL13B knockdown (KD) decreased the length of cilia and enhanced TGFβ1-induced EMT, evidenced by morphology change and a globally upregulated EMT-related gene expression in RPEs. Moreover, ARL13B KD enhanced TGFβ1-induced RPE proliferation and migration. Consistently, ARL13B KD promoted PVR formation *in vivo*. Mechanistically, ARL13B KD enhanced TGFβ1 signaling by increasing the phosphorylation and expression of Smad3.

**Conclusion:**

This study demonstrated a crucial role of ARL13B on TGFβ1-induced RPE EMT, highlighting the importance of ARL13B in PVR formation.

## 1 Introduction

Proliferative vitreoretinopathy (PVR) is a serious complication of rhegmatogenous retinal detachment (RRD) or retinal surgery. The abnormal scar tissue within the proliferative membrane can contract and pull on the retina, resulting in recurrent detachment and leading to vision loss (Pennock et al., 2014; Machemer et al., 1983). Surgery remains the most effective treatment for PVR; however, recurrence is common. Therefore, it is essential to understand the pathological mechanisms of PVR and identify novel drug targets to prevent its formation.

PVR development is a fibrotic process characterized by the formation of proliferative membranes composed of various fibrotic cellular elements. Whereas retinal pigment epithelial (RPE) cells are the most predominant component, the epithelial-mesenchymal transition (EMT) of RPE cells is the key pathological process in the development of PVR. RRD or retinal surgery causes the breakdown of the blood-retina barrier and the release of inflammatory cytokines and growth factors (e.g., TGF-β, PDGF, and VEGF) (Garweg et al., 2013; Govers et al., 2023). When stimulated by these cytokines or factors, RPE cells undergo EMT: lose their epithelial characteristics, including polarity and cell-cell adhesion, and acquire mesenchymal traits. These include the expression of mesenchymal markers such as fibronectin and N-cadherin, as well as a transition to a spindle-shaped morphology with enhanced migratory capacity (Chiba, 2014). Meanwhile, the transdifferentiated RPE cells gain the ability to secrete extracellular matrix components such as fibronectin to form fibrous membranes. Upon EMT, RPE cells can further differentiate into α-smooth muscle actin (α-SMA) expressing myofibroblasts that can promote the contraction of fibrous membranes, resulting in recurrent retinal detachment (Tamiya et al., 2010; Stocks et al., 2001).

ARL13B (ADP-ribosylation factor-like 13B) is a member of the ARF family of regulatory GTPases of the RAS superfamily. It is highly enriched in the membrane of non-motile cilia, also known as primary cilia, and plays a crucial role in the maintenance of primary cilia structure and function (Li et al., 2010; D'Souza-Schorey and Chavrier, 2006). ARL13B regulates intraflagellar transport (IFT), a process required for ciliogenesis and cilia maintenance, by interacting with multiple cilia proteins, including the exocyst, tubulin, UNC119, and INPP5E, a phosphatidylinositol phosphatase (Seixas et al., 2016; Revenkova et al., 2018; Zhang et al., 2016; Humbert et al., 2012). Loss of ARL13B function results in shortened and misshapen cilia, affecting its ability to properly respond to extracellular signals such as Hedgehog signaling (Li et al., 2010; Caspary et al., 2007; Gigante et al., 2020). ARL13B can also act as a guanine nucleotide exchange factor (GEF) for ARL3, another ciliary ARF-like (ARL) protein (Zhang et al., 2016; Gotthardt et al., 2015). This function is crucial for cilia-related trafficking and protein targeting, especially in photoreceptor cells and other ciliated cell types (Gotthardt et al., 2015).

Here, we detected a significant reduction of ARL13B protein levels in mesenchymal transitioned cells in the PVR membrane from RRD patients. Moreover, we found that loss of ARL13B enhanced TGFβ1-induced EMT of RPE cells and PVR formation. Mechanistically, we found that loss of ARL13B increases phosphorylation and protein levels of Smad3. Our findings highlight the critical role of ARL13B in PVR pathogenesis and suggest potential therapeutic strategies targeting ARL13B.

## 2 Materials and methods

### 2.1 PVR clinical specimens

The study with human PVR tissues was approved by the ethics committee of Zhongshan Ophthalmic Center (2020KYPJ016) and followed the Declaration of Helsinki. All patients provided informed consent. Proliferative membranes were discarded tissues during vitrectomy from PVR patients following RRD, immediately fixed in 4% paraformaldehyde for 24 h, and followed by immunofluorescence and analysis.

### 2.2 Cell culture and reagents

The human RPE cell line (ARPE-19) and the HEK-293T cell line were obtained from the American Type Culture Collection (ATCC, Manassas, VA, United States). Cells were cultured in DMEM/F12 or DMEM medium (Gibco, United States) with 10% fetal bovine serum and 1% Penicillin-Streptomycin at 37 °C in 5% CO2. 10 ng/mL TGF-β1 (Novoprotein, Suzhou, China) was used to induce EMT for 48 h (Ma et al., 2023). All cell lines were validated by STR analysis and tested for *mycoplasma* contamination.

### 2.3 Stable cell line establishment

Lentivirus was packaged in 293T cells by transfecting with psPAX2, pMD2. G plasmids, and shRNAs targeting ARL13B or a scramble control (Transheepbio, Suzhou, China) with TurboFect (Thermo, United States), collected, and used to infect ARPE-19 cells in 10 μg/mL Polybrene (Solarbio, Beijing, China). 48 h later, 2 μg/mL puromycin (Solarbio, Beijing, China) was applied to select infected cells.

### 2.4 EdU staining

Cells were seeded in 96-well plates at a density of 1 × 10^4^ and treated with TGF-β1. After 2 h of EdU incubation (Ribobio, Guangzhou, China), cells were fixed, permeabilized, and stained with Apollo and Hoechst. EdU-positive cells were visualized using a fluorescence microscope (Nikon, Japan) and quantified using ImageJ (NIH, United States) by calculating the percentage of EdU-positive cells (EdU stained/Hoechst stained × 100%).

### 2.5 Scratch assay

Cells were seeded in 6-well plates at a density of 0.6 × 10^6^. A 200 µL plastic pipette tip was used to create a scratch at 80% confluence. Scratch images at 0 and 72 h with TGF-β1 were captured using an optical microscope (Nikon). The scratched areas were measured using ImageJ software (NIH, United States), and the wound closure rate was calculated as (A0 − A72)/A0 × 100%, where A0 is the area at 0 h and A72 is the area at 72 h.

### 2.6 Transwell assay

200 µL of cells at a density of 1 × 10^5^ with TGF-β1 were seeded in the transwell insert (Corning). The lower chambers were filled with 600 µL medium containing 10% FBS. After 24 h, cells that migrated to the lower chamber were fixed, stained with crystal violet (Solarbio, China), and quantified using ImageJ. Migrated cells were imaged using an optical microscope (Nikon).

### 2.7 Immunofluorescence staining

Coverslips with cells, PVR clinical specimens, or mouse eye sections were fixed, blocked, and incubated with primary antibodies overnight at 4 °C. Followed by secondary antibodies (anti-mouse Alexa 488, 555; anti-rabbit Alexa 555, 647; Thermo Fisher Scientific, United States) and DAPI staining. Images were captured using a confocal microscope (Zeiss, Germany). Primary antibodies included ARL13B (1:200, 17711-1-AP, Proteintech, China), α-SMA (1:200, A2547, Siama, United States), and Smad3 (1:200, 9523, CST, United States).

### 2.8 Western blotting

Total proteins were extracted using lysis buffer (Keygentec, China) and quantified via BCA assay (Thermo Fisher Scientific, United States). After SDS-PAGE (8% or 10%), proteins were transferred to PVDF membranes (Millipore, United States), blocked, and incubated with primary antibodies overnight at 4 °C, followed by HRP-conjugated secondary antibodies (CST, United States). Chemiluminescent substrate (Millipore, United States) was used for detection, and blots were visualized. Primary antibodies: ARL13B (1:1000, 17711-1-AP, Proteintech), Fibronectin (1:1000, 26836, CST, United States), Vimentin (1:1000, 5741, CST, United States), ZO-1 (1:10000, 33-9100, Thermo, United States), phospho-Smad3 (1:1000, 9520, CST, United States), Smad3 (1:1000, 9523, CST, United States), and GAPDH (1:1000, 2118, CST, United States). Band intensity was analyzed using ImageJ (NIH).

### 2.9 Real-time PCR

Total RNA was extracted according to the manufacturer’s instructions (Esscience, China) and synthesized to cDNA (Takara, Japan). Real-time PCR was performed using SYBR Green Master Mix (Roche, Switzerland) on the Roche 480 system (Switzerland). Primer sequences are listed in Supplementary Table S1. Relative gene expression was calculated using the 2^−ΔΔCt^ method, with GAPDH as an internal control.

Total RNA from cells was isolated (Esscience, China).

### 2.10 RNA-sequencing

Total RNA from cells was isolated (Esscience, China). Enriched for mRNA and sequenced on the Illumina NovaSeq 6000 platform. FPKM values were calculated using StringTie, and differentially expressed genes were identified with the edgeR package. All RNA-seq samples were processed and sequenced in the same batch, using identical protocols and reagents. Genes with a log2 (fold change) > 0.5 and adj. p.value <0.05 were considered statistically significant. Gene set enrichment analysis (GSEA) was conducted using DAVID software.

### 2.11 Establishment of PVR mouse model

All animal procedures were approved by the Animal Care and Use Committee of Sun Yat-sen University (2020-092) and followed the ARVO Statement for the Use of Animals in Ophthalmic and Vision Research. C57BL/6 J mice (6–8 weeks, 30 ± 10 g) from Charles River Laboratories (Beijing Vital River Laboratory Animal Technology Co., Ltd.) were used. The fundus was checked before the experiments. Mice were anesthetized, and pupils were dilated before intravitreal injection of 1 × 10^4^ ARPE-19 cells. Fundus images were captured with a retinal imaging system (Phoenix Micron IV). PVR stages were assessed at 3, 7, and 14 days using Fastenberg’s classification: stage 0: no proliferative response; stage 1: intravitreal proliferation; stage 2: focal traction with localized vascular changes; stage 3:localized detachments; stage 4:extensive retinal detachment; stage 5: total retinal detachment (Fastenberg et al., 1982).

### 2.12 Hematoxylin-eosin staining

Mouse eyeballs were fixed, embedded, and sectioned into 5 µm slices. After deparaffinization and rehydration, tissues were stained with hematoxylin, differentiated in hydrochloric ethanol, and stained with eosin. Samples were dehydrated, cleared in xylene, and imaged using a light microscope. (Nikon).

### 2.13 Statistical analyses

Statistical analyses were performed using GraphPad Prism 9.0 (GraphPad Software, Inc.). Comparisons were made using the Mann-Whitney U test and the unpaired Student’s t-test. Data are presented as mean ± standard deviation (SD) from at least three independent experiments, with *p* < 0.05 considered statistically significant.

## 3 Results

### 3.1 Reduced ARL13B protein is associated with RPE-EMT in PVR

**FIGURE 1:**
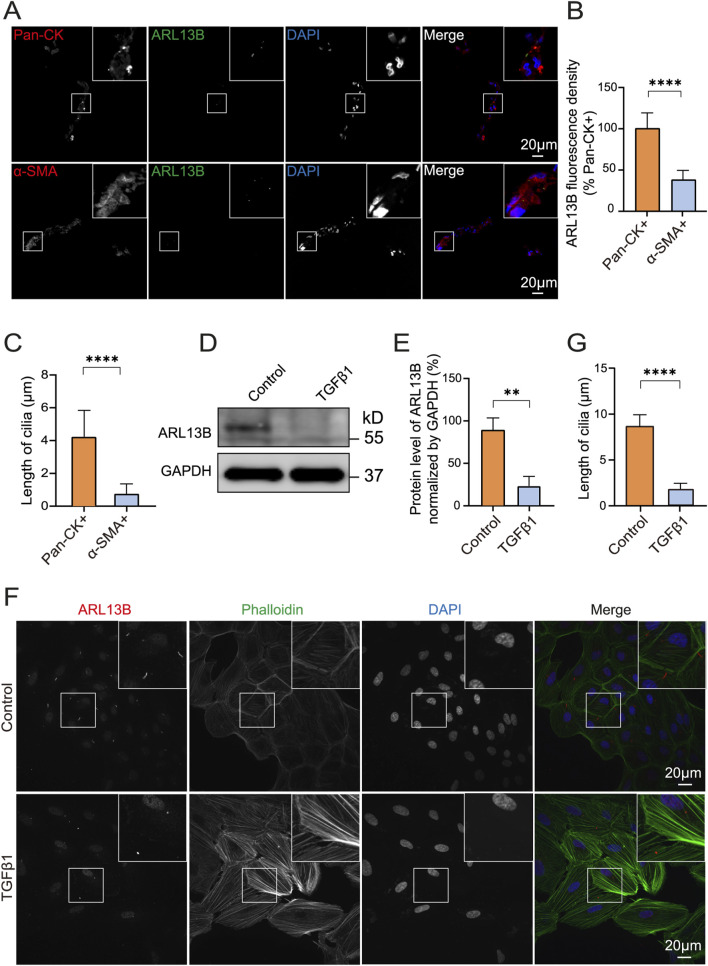
Reduced ARL13B protein levels in PVR and during the EMT of ARPE-19 cells. **(A)** Immunofluorescence staining of primary cilia protein ARL13B in PVR clinical specimens. ARL13B (green), Pan-CK (red), α-SMA (red), and DAPI (blue). **(B,C)** Quantification of ARL13B expression and cilia length (μm) in pan-CK+ and α-SMA + cells. **(D)** Western blot of ARL13B in ARPE-19 cells with TGF-β1treatment. **(E)** Statistical analysis of ARL13B expression in **(D)**. **(F)** Immunofluorescence staining of ARL13B in ARPE-19 cells with TGF-β1 treatment. Cilia (ARL13B, red), cytoskeleton (Phalloidin, green), and nuclei (DAPI, blue). **(G)** Quantification of the cilia length (μm) in **(F)**. Data are represented as mean ± SD. n = 3, **p < 0.01, ****p < 0.0001. Scale bar: 20 μm.

After RRD, detached RPE migrates to epiretinal or subretinal and becomes the major cell component of the proliferative membrane, where it undergoes EMT with molecular features switching from epithelial to mesenchymal (Chiba, 2014). To investigate the expression of ciliary protein ARL13B in PVR specimens, we performed immunofluorescence staining and found that ARL13B expression was significantly lower in α-SMA + cells as compared to the Pan-CK + cells (Figures 1A,B). Accordingly, shortened primary cilia were found in α-SMA + cells as compared to the Pan-CK + cells (Figure 1A,C). To further investigate the role of ARL13B in EMT, we used TGF-β1 to induce EMT of ARPE-19 *in vitro*. The Western blot showed a significant reduction of ARL13B with TGF-β1 treatment in ARPE-19 cells (Figures 1D,E), accompanied by shortened primary cilia in immunofluorescence staining (Figures 1F,G). These results were further confirmed by using iPSC-RPE cells (Supplementary Figure S1). Together, these findings suggest that reduced ciliary protein ARL13B RPE is associated with RPE-EMT during PVR.

### 3.2 Loss of ARL13B leads to cilia deficiency in RPE cells

**FIGURE 2:**
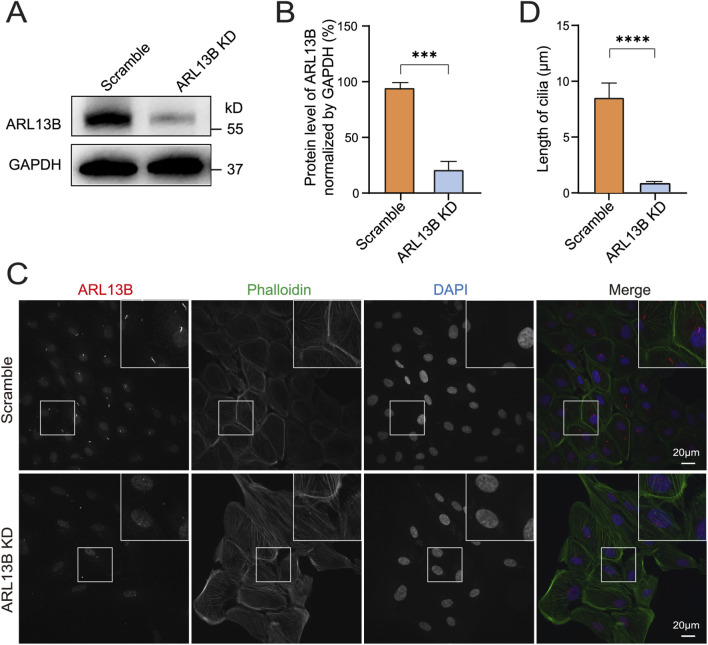
Loss of ARL13B leads to cilia deficiency. **(A)** Western blot of ARL13B in the scramble and ARL13B KD groups. **(B)** Quantification of ARL13B expression in **(A)**. **(C)** Immunofluorescence staining of primary cilia in scramble and ARL13B KD groups. Cilia (ARL13B, red), cytoskeleton (Phalloidin, green), nuclei (DAPI, blue). **(D)** Quantification of cilia length (μm) in **(C)**. Data are represented as mean ± SD. n = 3, ***p < 0.001, ****p < 0.0001, Scale bar: 20 μm.

ARL13B is an essential component of primary cilia and is involved in cilia structure maintenance in different cell types (Larkins et al., 2011). To further explore the effect of ARL13B on the cilia structure of RPE cells, we knocked down ARL13B in ARPE-19 cells by using ARL13B shRNA lentivirus. Western blot confirmed a robust reduction of ARL13B in knockdown (ARL13B KD) cells compared to scramble shRNA controls (Figures 2A,B). Notably, immunofluorescence staining demonstrated a significant shortening of primary cilia in ARL13B KD cells (Figures 2C,D), indicating that loss of ARL13B can lead to cilia deficiency in ARPE-19 cells.

### 3.3 Loss of ARL13B enhances the EMT of RPE cells

**FIGURE 3:**
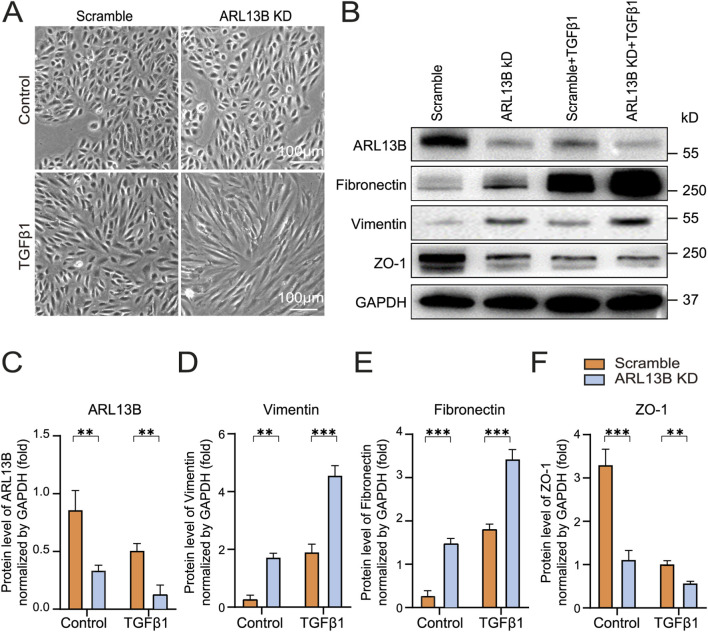
Effects of ARL13B on TGF-β1-induced EMT in RPE Cells. **(A)** Morphological changes in the scramble and ARL13B KD groups with or without TGF-β1. **(B)** Western blots of EMT markers (Fibronectin, Vimentin, ZO-1) in scramble and ARL13B KD groups with or without TGF-β1. **(C–F)** Quantification of protein expression in **(B)**. Data are represented as mean ± SD. n = 3, *p < 0.05, **p < 0.01, ***p < 0.001. Scale bar: 100 μm.

To explore the role of ARL13B in TGF-β1-induced EMT *in vitro*, we first investigated morphological changes of ARL13 B-deficient RPE cells. As the phase image results showed, the loss of ARL13B alone resulted in a cellular morphological change from oval to spindle-shaped. This morphological change was more pronounced upon TGF-β1 (Figure 3A), indicating that loss of ARL13B facilitates the transition from an epithelial to a mesenchymal phenotype for RPE cells. Consistently, Western blot showed that loss of ARL13B led to upregulation of mesenchymal markers fibronectin and vimentin, accompanied by downregulation of the epithelial marker ZO-1, especially under the treatment of TGF-β1. (Figures 3B–F). These findings suggest that loss of ARL13B promotes EMT in RPE cells.

**FIGURE 4:**
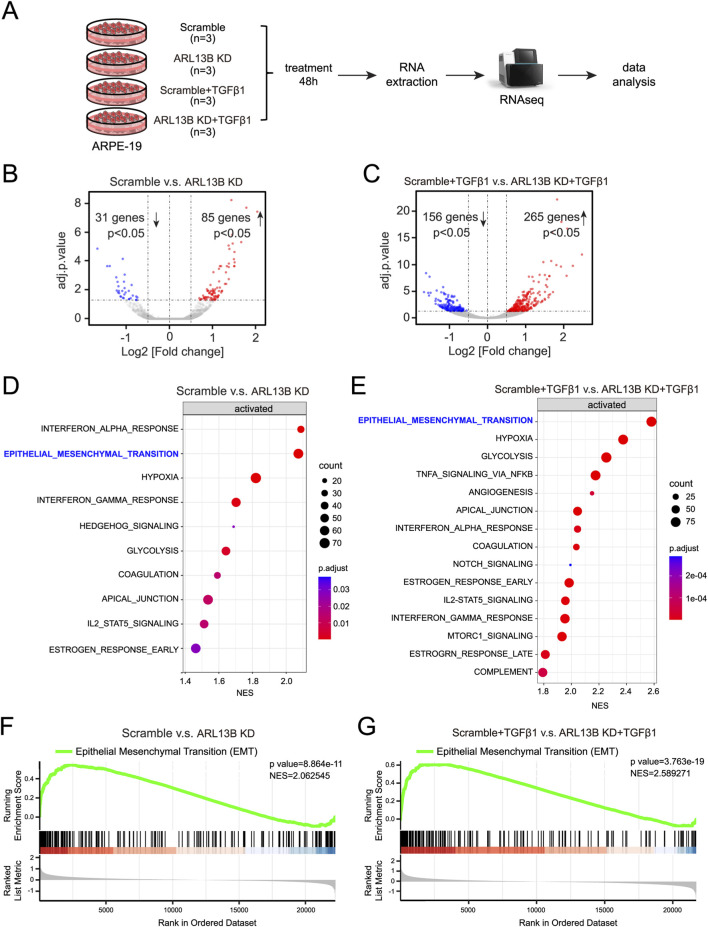
Bulk RNA-seq analysis of the effect of ARL13B on TGF-β1-induced EMT of RPE Cells. **(A)** The experimental workflow. **(B,C)** Volcano plots showing differentially expressed genes between the scramble and ARL13B KD groups **(B)** without or **(C)** with TGF-β1. **(D,E)** GSEA terms for differentially expressed genes in **(D)** basal conditions and **(E)** TGF-β1-treated cells. **(F,G)** GSEA reveals EMT-related gene enrichment scramble and ARL13B KD groups without **(F)** and with **(G)** TGF-β1.

To gain a view of the global impact of ARL13B on the EMT of RPE cells, we conducted a bulk RNA-seq (Figure 4A). The volcano plots showed that compared to scramble controls, ARL13B knockdown cells had 85 upregulated and 31 downregulated differentially expressed genes (Figure 4B). The number of upregulated and downregulated differentially expressed genes increased to 265 and 156, respectively, upon TGF-β1 treatment (Figure 4C). GSEA analysis of the differentially expressed genes showed EMT as the top significantly enriched processes in ARL13B knockdown cells with or without TGF-β1 treatment (Figures 4D,E). The GSEA further confirmed a global activation of EMT-related genes in ARL13B KD cells, especially with TGF-β1 (Figures 4F,G). These findings indicate that loss of ARL13B enhances the EMT in RPE cells.

### 3.4 Loss of ARL13B promotes the proliferation of RPE cells

**FIGURE 5:**
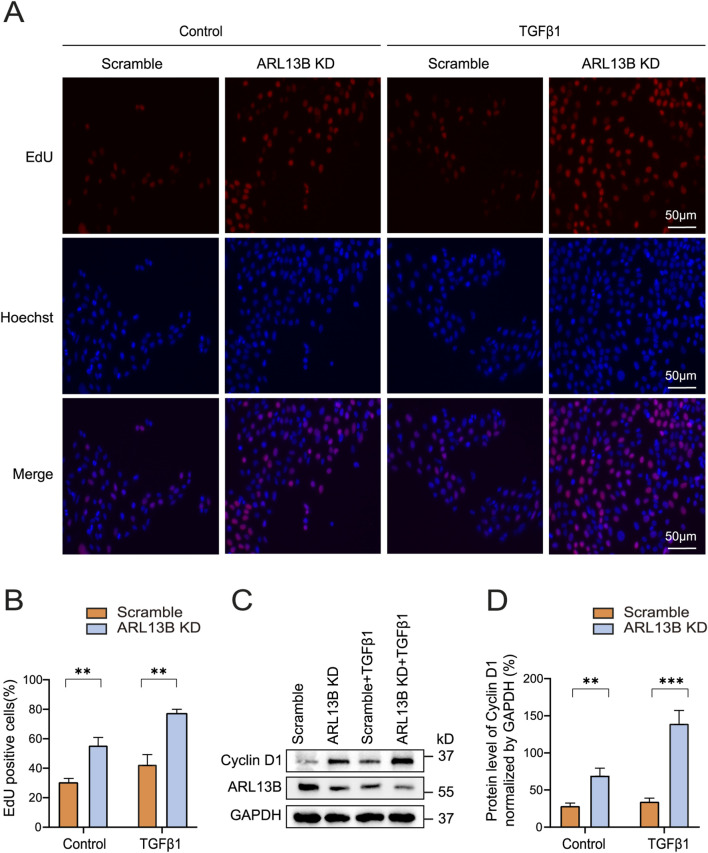
Effects of ARL13B on ARPE-19 cell proliferation. **(A)** EdU stains of proliferation in the scramble and ARL13B KD group with or without TGF-β1. EdU (red), Hoechst (blue). **(B)** Quantification of the EdU + cells in **(A)**. **(C)** Western blot of Cyclin D1 in the scramble and ARL13B KD cells with or without TGF-β1. **(D)** Statistical analysis of Cyclin D1 expression in **(C)**. Data are represented as mean ± SD. n = 3, **p < 0.01, ***p < 0.001. Scale bar: 50 μm.

The formation of proliferative membranes relies on RPE cell proliferation (Chiba, 2014). To investigate the role of ARL13B in RPE proliferation, we conducted an EdU assay. The results showed that ARL13B knockdown alone increased the number of EdU-labeled cells, and this effect was further enhanced upon TGF-β1 treatment (Figures 5A,B), indicating that ARL13B loss promotes RPE cell proliferation both in the absence and presence of TGF-β1. We further examined the expression of cyclin D1, a key regulatory protein controlling the transition from the G1 phase to the S phase. The Western blot revealed that ARL13B knockdown increased cyclin D1 expression, with or without TGF-β1 treatment (Figures 5C,D). Those results suggest that loss of ARL13B promotes RPE proliferation at least partially by upregulating cyclin D1 expression.

### 3.5 Loss of ARL13B accelerates the migration of RPE cells

**FIGURE 6:**
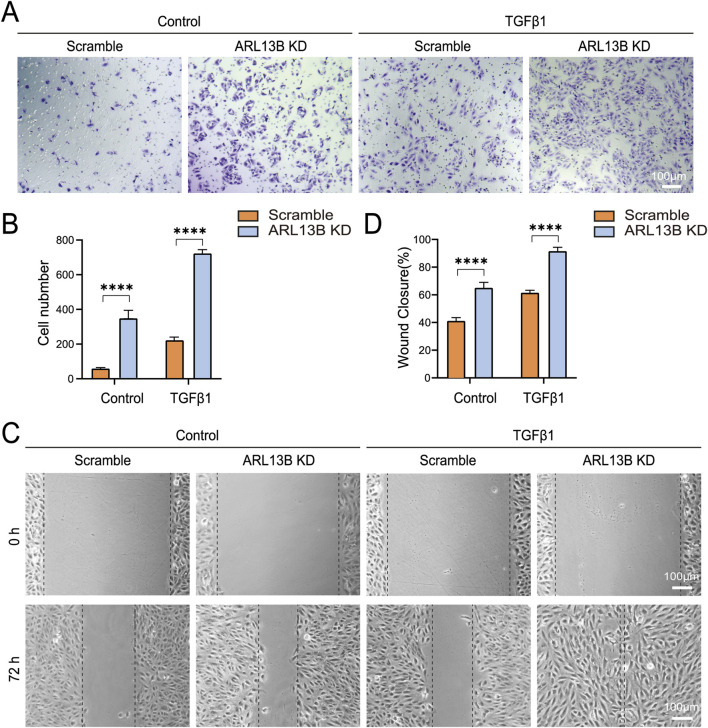
Effects of ARL13B on ARPE-19 cell migration. **(A)** Transwell assay in the scramble and ARL13B KD cells with or without TGF-β1. **(B)** Quantification of cells in **(A)**. **(C)** Scratch assay in the scramble and ARL13B KD cells with or without TGF-β1 at 0 h and 72 h. **(D)** Quantification of the wound closure rate (%). Data are represented as mean ± SD. n = 3, ****p < 0.0001. Scale bar: 100 μm.

To investigate the role of ARL13B in RPE migration, we performed transwell and scratch assays. The transwell assay showed that ARL13B knockdown enhanced RPE cell migration, particularly upon TGF-β1 treatment (Figures 6A,B). Consistently, the scratch assay showed that ARL13B knockdown accelerated wound closure compared to the scramble group, both in the absence and presence of TGF-β1 (Figures 6C,D).

**FIGURE 7:**
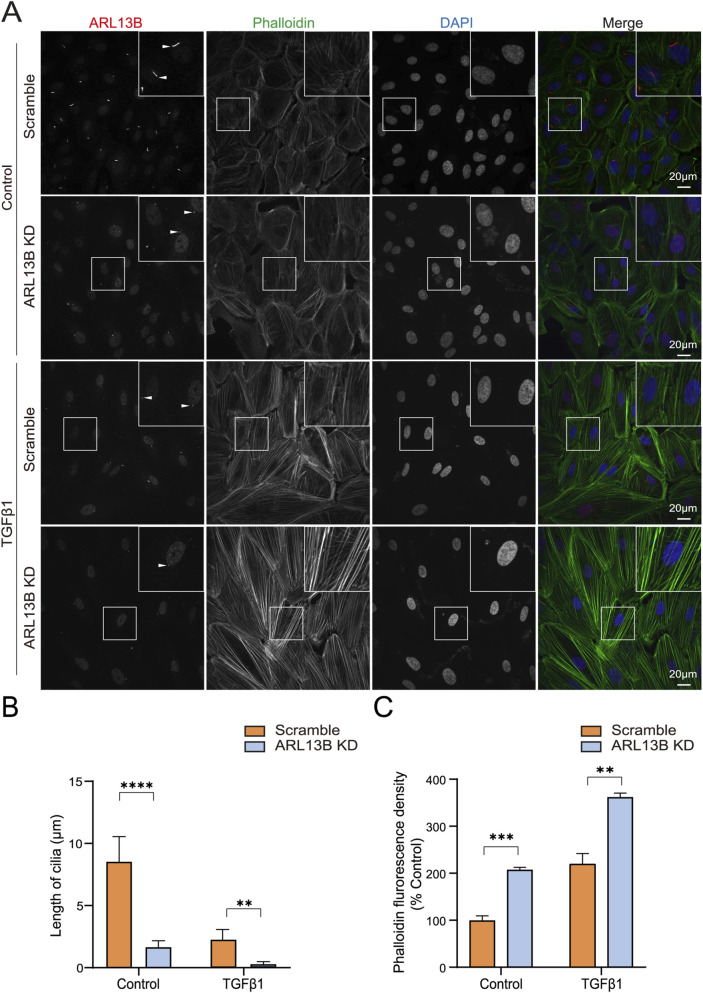
Effects of ARL13B on ARPE-19 cell cytoskeleton remodeling. **(A)** Immunofluorescence staining of ARL13B in the scramble and ARL13B KD cells with or without TGF-β1. Cilia (ARL13B, red), cytoskeleton (Phalloidin, green), and nuclei (DAPI, blue). **(B,C)** Quantification of cilia length (μm) **(B)** and Phalloidin fluorescence density **(C)**. Data are represented as mean ± SD. n = 3, **p < 0.01, ***p < 0.001, ****p < 0.0001. Scale bar: 20 μm.

Cell migration relies heavily on a dynamic rearrangement of the actin cytoskeleton (Thapa et al., 2023). To investigate the cytoskeletal dynamics, we visualized the F-actin (filamentous actin) using phalloidin staining. The immunofluorescence staining revealed increased phalloidin upon ARL13B knockdown, with more pronounced changes in TGF-β1 treatment (Figures 7A,C). This cytoskeletal remodeling was correlated with the shortening of cilia length (Figure 7A,B). These data indicate that the loss of ARL13B enhances the migration of RPE cells.

### 3.6 Loss of ARL13B enhances the progression of PVR *in vivo*

**FIGURE 8:**
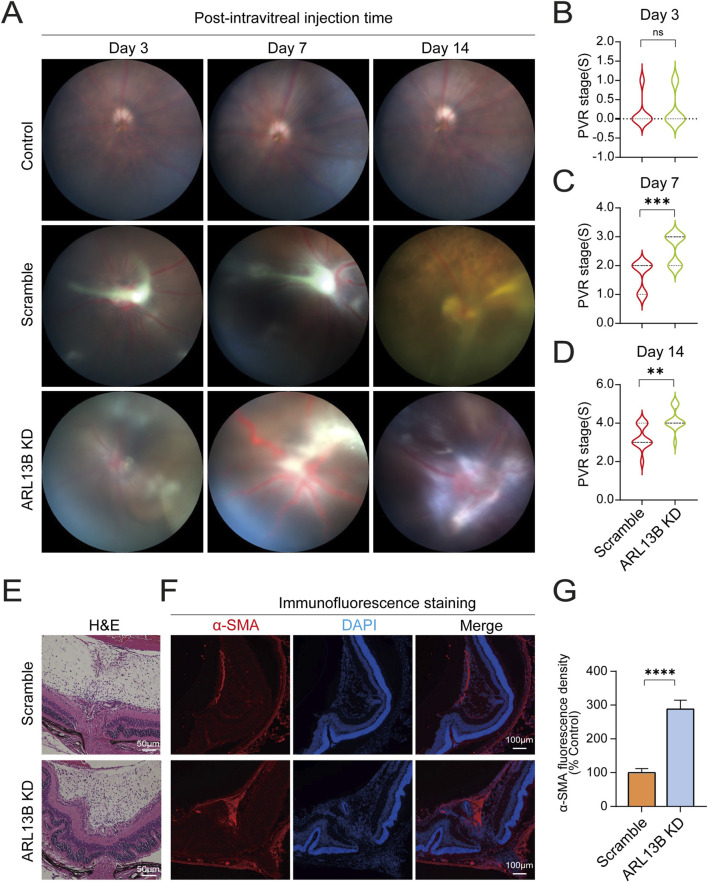
Effects of ARL13B on PVR formation *in vivo*. **(A)** The mouse fundus after intravitreal injection with scramble and ARL13B KD cells. **(B–D)** Statistical analysis of mice PVR stages at day 3 **(B)**, day 7 **(C)**, and day 14 **(D)** after intravitreal injection. **(E)** H&E stains of eye sections on day 14. **(F)** Immunofluorescence staining of α-SMA (red) of eye sections on day 14. Nuclei, (DAPI, blue). **(G)** Quantification of α-SMA fluorescence density in **(F)**. Data were analyzed by the Mann-Whitney U test (n = 12). ns, no significance, **p < 0.01, ***p < 0.001. Scale bar: 100 μm.

Previously, we have shown that loss of ARL13B promotes EMT, proliferation, and migration *in vitro*. To further investigate the role of ARL13B in PVR formation, we injected ARL13B KD and scrambled ARPE-19 cells into the vitreous of mice as described in our previous study (Ma et al., 2023). We then assessed PVR progression via fundus photography with Fastenberg classification on days 3, 7, and 14. On day 3, both ARL13B KD and scrambled groups showed similar cell distribution across the retinal surface (Figures 8A,B). On day 7, retinal detachment was observed in both groups, but more severe in the ARL13B KD group, the majority of which progressed to stage 3, while scrambled controls remained at stage 2 (Figures 8A,C). On day 14, the ARL13B KD group showed more extensive detachment, reaching stages 4 or 5, compared to stages 3 or 4 for the majority of scrambled controls. (Figures 8A,D). Histological analysis on day 14 confirmed more severe retinal detachment and increased α-SMA expression in the ARL13B KD group (Figures 8E,F). Together, these findings indicate that loss of ARL13B promotes PVR formation.

### 3.7 Loss of ARL13B enhances EMT by increasing Smad3 expression in RPE cells

**FIGURE 9:**
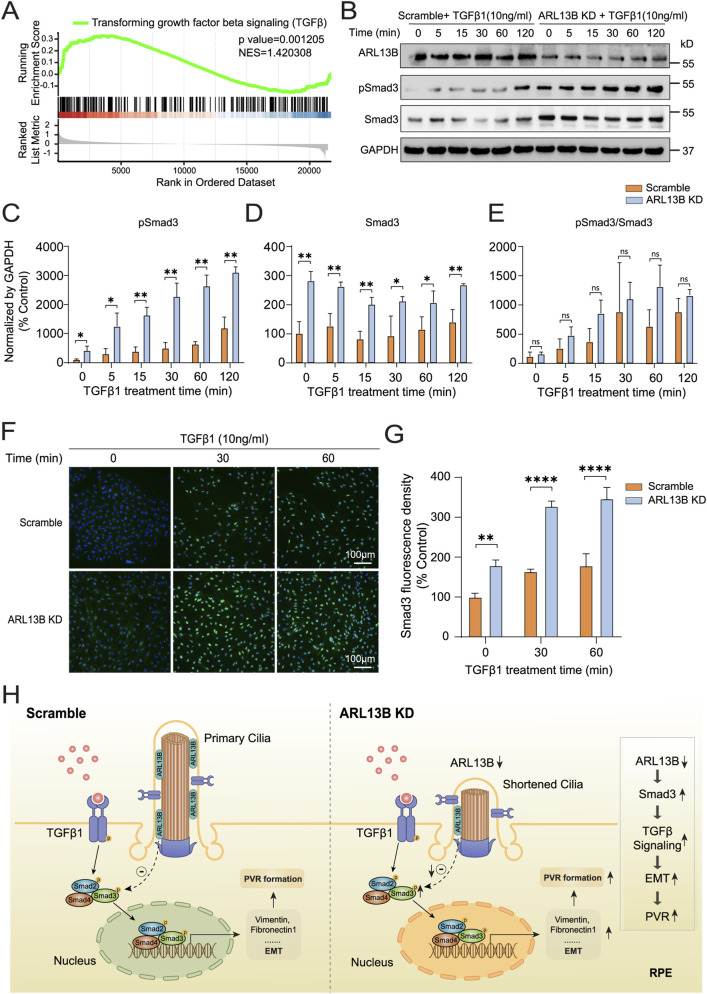
Effects of ARL13B on TGF-β1 signaling in RPE cells. **(A)** GSEA terms of TGF-β1 genes in the scramble and ARL13B KD cells with TGF-β1. **(B)** Western blot of ARL13B, Smad3, and pSmad3 in the scramble and ARL13B KD group with TGF-β1 at different times. **(C–E)** Statistical analysis of the protein expression. **(C)** pSmad3, **(D)** Smad3, **(E)** the ratio of pSmad3/Smad3. **(F)** Immunofluorescence staining of Smad3 (green) in the scramble and ARL13B KD cells with TGF-β1 at different times, nuclei with DAPI (blue). **(G)** Quantification of the Smad3 fluorescence density in **(F)**. **(H)** A diagram that shows the mechanism of the effect of ARL13B on TGFβ1-induced EMT of RPE in PVR. Data are represented as mean ± SD. n = 3, ns, no significance, *p < 0.05, **p < 0.01, ****p < 0.0001. Scale bar:100 μ.m.

To explore the molecular mechanism by which ARL13B regulates EMT, we further analyzed bulk RNA-seq data described earlier in results 3.3, and the GSEA analysis found that ARL13B knockdown significantly upregulated TGF-β1 signaling (Figure 9A). To further investigate the effect of ARL13B on TGF-β1 signaling, we first examined the canonical TGF-β1 signaling pathway by checking phosphorylated Smad3 (pSmad3) levels. As the Western blot results showed, pSmad3 levels increased starting at 5 min after TGF-β1 treatment, with significantly higher expression in the ARL13B KD group, indicating enhanced Smad3 activation (Figures 9B,C). However, there was no significant difference in the ratio of phosphorylated and total Smad3 between the two groups (Figure 9E). Notably, total Smad3 levels were elevated in the ARL13B KD group (Figures 9B,D), suggesting that increased pSmad3 resulted from elevated total Smad3. Consistently, the immunofluorescence staining showed increased nuclear translocation of Smad3 in the ARL13B KD group in both the absence and presence of TGF-β1 (Figures 9F,G). Meanwhile, we also detected the pSmad3 by immunofluorescence staining, the result showed that ARL13B knockdown enhanced nuclear translocation of Smad3 (Supplementary Figure S2). In parallel, we examined the involvement of the non-canonical TGF-β1 signaling pathway, like the ERK pathway. Western blot analysis revealed no significant differences in total ERK and phosphorylated ERK (p-ERK) levels between ARL13B KD and scramble cells, and the p-ERK/ERK ratio remained unchanged (Supplementary Figure S3). These findings suggest that the ERK pathway does not participate in the EMT induced by ARL13B deficiency. To further explore the regulation of ARL13B on Smad3, we performed real-time PCR analysis, which revealed that ARL13B deficiency did not affect Smad3 mRNA expression (Supplementary Figure S4). These results imply that ARL13B may regulate Smad3 levels at the protein translation or stability instead of the transcriptional level. Given that the cilia signaling, such as the Hedgehog pathway, is known to be involved in the EMT (Wang et al., 2024), we next investigated whether the Hedgehog pathway contributes to the ARL13B deficiency–mediated enhancement of EMT. Western blot analysis revealed no significant changes in PTCH2 and SHH protein expression, indicating that ARL13B deficiency regulates EMT independently of cilia-associated signals such as the Hedgehog pathway (Supplementary Figure S5). Taken together, loss of ARL13B may enhance RPE-EMT by upregulating Smad3 expression through post-transcriptional regulation.

## 4 Discussion

PVR is the major cause of surgical failure in RRD. The contraction of proliferative membranes in PVR can cause re-detachment of the retina, leading to vision loss (Chiba, 2014; Lumi et al., 2015). The EMT of RPE cells is the core pathology of PVR, yet its regulation mechanism remains incompletely understood. In this study, we found unexpectedly that the protein level of ARL13B, a primary cilia protein, was significantly reduced in the mesenchymal-transited cells in PVR specimens from RRD patients. We further demonstrated that loss of ARL13B enhanced the proliferation, migration, and EMT of ARPE-19 cells, and exacerbated PVR progression *in vivo*. Mechanistically, we found that loss of ARL13B increased Smad3 phosphorylation and nuclear translocation by elevating Smad3 protein level (Figure 9H).

ARL13B is a small GTPase localized to primary cilia. It plays an important role in maintaining cilia structure and signaling transduction. Dysregulation of ARL13B has been linked to ciliopathies such as Joubert syndrome (Cevik et al., 2010). In this study, we found a reduction of ARL13B and cilia shortening during EMT of PVR specimens, and loss of ARL13B enhanced TGF-β1-induced EMT of RPE cells. Notably, TGF-β1 treatment resulted in a significant reduction of ARL13B in RPE cells. In line with that, reduced ARL13B expression was also observed in TGF-β1-induced EMT in alveolar epithelial cells (Ni et al., 2024). Moreover, loss of ARL13B in renal epithelial cells can also lead to ciliary defects and trigger EMT (Han et al., 2018). Together, these findings suggest that ARL13B plays an essential role in the regulation of EMT crossing different cell types.

The proliferation of RPE cells is an important pathogenic event in the development of PVR. Our EdU incorporation and Cyclin D1 expression data revealed that loss of ARL13B promoted RPE cell proliferation. It is known that ciliogenesis is incompatible with cell cycle progression, as ciliogenesis shares the centriole with the mitotic spindle (Al Jord et al., 2017). Therefore, a cell must resorb its cilium before using the centrosome for cell division. Thus, ciliary disassembly is required for cell cycle re-entry. Since our results showed that loss of ARL13B led to cilia deficiency, its effect on proliferation is likely via disassembling the cilia of RPE cells. In line with this, cilia-deficient cancer cells also showed increased proliferation (Li et al., 2023).

Migration of RPE is also essential for the formation of proliferative membranes in PVR (Garweg et al., 2013). We found that ARL13B knockdown enhanced RPE migration in both transwell and scratch assays. Similarly, loss of ARL13B also impaired neuronal migration in the cortex (Higginbotham et al., 2012), suggesting ARL13B is implicated in the migration of different cell types. We further observed enhanced cytoskeletal remodeling in ARL13B-deficient RPE cells, implicating ARL13B in regulating cytoskeletal architecture. As the cytoskeleton governs polarity and membrane protrusions, its remodeling is essential for migration (Thapa et al., 2023; Dobbelaere, 2023). Therefore, ARL13B loss may promote RPE migration via cytoskeletal remodeling.

TGF-β1 is the most critical factor inducing EMT of RPE cells during the development of PVR. Binding to its receptors on the cell surface, TGF-β1 activates either the canonical (Smad-dependent) or non-canonical (Smad-independent) pathways to promote the expression of EMT-related genes (Xu et al., 2009; Derynck and Zhang, 2003). Our RNA-seq analysis revealed that loss of ARL13B activated the TGF-β signaling. To investigate the underlying mechanisms, we first examined the canonical pathway by checking Smad3 phosphorylation levels. We found that loss of ARL13B increased both phosphorylation and total levels of Smad3. Meanwhile, we investigated the non-canonical TGF-β1 pathway ERK and observed no significant differences between ARL13B KD and scramble cells, indicating that ARL13B deficiency–induced RPE-EMT is mediated through the regulation of Smad3 expression in the TGF-β1 canonical signaling pathway. While previous studies have not directly linked ARL13B to the Smad pathway, growing evidence suggests that primary cilia are critical in coordinating TGF-β/Smad signaling (Christensen et al., 2017; Yu et al., 2024). Moreover, TGF-β receptors and Smad components have also been found to localize within the cilium (Labour et al., 2016). It is known that cilia signaling, like the Hedgehog pathway, is involved in multiple cellular biological processes, including EMT (Wang et al., 2024). We further investigated whether this pathway participates in the regulation of EMT by ARL13B. Western blot analysis revealed no significant changes in SHH or PTCH2 expression. Similarly, Gigante et al. demonstrated that defects in primary cilia do not necessarily correlate with changes in Hedgehog signaling (Gigante et al., 2020). These results suggest that ARL13B deficiency promotes RPE-EMT independently of the Hedgehog pathway. To further explore the mechanism of ARL13B regulation of Smad3, we performed real-time PCR and observed no significant changes at the mRNA level of Smad3, suggesting that ARL13B may regulate Smad3 via post-transcriptional mechanisms. Taken together, our findings indicate that ARL13B deficiency enhances RPE-EMT primarily through Smad3-dependent mechanisms. Whether other TGF-β1 non-canonical signaling pathways and cilia signaling, such as the Wnt pathway, need to be further investigated.

## 5 Conclusion

In summary, our study demonstrates that loss of the ciliary protein ARL13B promotes TGF-β1-induced EMT, proliferation, and migration of RPE cells, as well as PVR progression, by upregulating Smad3 expression. Our findings highlight the critical role of ARL13B in PVR pathogenesis and suggest potential therapeutic strategies targeting ARL13B.

## Data Availability

All data generated or analysed during this study are included in this published article, and the RNA sequence data reported in this paper have been deposited in the Genome Sequence Archive (Genomics, Proteomics & Bioinformatics 2025) in National Genomics Data Center (Nucleic Acids Res 2025), China National Center for Bioinformation / Beijing Institute of Genomics, Chinese Academy of Sciences (GSA-Human: HRA014635).

## References

[B1] Al Jord A. Shihavuddin A. Servignat D'aout R. Faucourt M. Genovesio A. Karaiskou A. (2017). Calibrated mitotic oscillator drives motile ciliogenesis. Science 358, 803–806. 10.1126/science.aan8311 28982797

[B2] Caspary T. Larkins C. E. Anderson K. V. (2007). The graded response to sonic hedgehog depends on cilia architecture. Dev. Cell 12, 767–778. 10.1016/j.devcel.2007.03.004 17488627

[B3] Cevik S. Hori Y. Kaplan O. I. Kida K. Toivenon T. Foley-Fisher C. (2010). Joubert syndrome Arl13b functions at ciliary membranes and stabilizes protein transport in *Caenorhabditis elegans* . J. Cell Biol. 188, 953–969. 10.1083/jcb.200908133 20231383 PMC2845074

[B4] Chiba C. (2014). The retinal pigment epithelium: an important player of retinal disorders and regeneration. Exp. Eye Res. 123, 107–114. 10.1016/j.exer.2013.07.009 23880527

[B5] Christensen S. T. Morthorst S. K. Mogensen J. B. Pedersen L. B. (2017). Primary cilia and coordination of receptor tyrosine kinase (RTK) and transforming growth factor β (TGF-β) signaling. Cold Spring Harb. Perspect. Biol. 9, a028167. 10.1101/cshperspect.a028167 27638178 PMC5453389

[B6] D'Souza-Schorey C. Chavrier P. (2006). ARF proteins: roles in membrane traffic and beyond. Nat. Rev. Mol. Cell Biol. 7, 347–358. 10.1038/nrm1910 16633337

[B7] Derynck R. Zhang Y. E. (2003). Smad-dependent and Smad-independent pathways in TGF-beta family signalling. Nature 425, 577–584. 10.1038/nature02006 14534577

[B8] Dobbelaere J. (2023). Cytoskeleton: the many flavors of cilia transition fibers. Curr. Biol. 33, R150–r153. 10.1016/j.cub.2023.01.021 36854274

[B9] Fastenberg D. M. Diddie K. R. Sorgente N. Ryan S. J. (1982). A comparison of different cellular inocula in an experimental model of massive periretinal proliferation. Am. J. Ophthalmol. 93, 559–564. 10.1016/s0002-9394(14)77369-6 7081354

[B10] Garweg J. G. Tappeiner C. Halberstadt M. (2013). Pathophysiology of proliferative vitreoretinopathy in retinal detachment. Surv. Ophthalmol. 58, 321–329. 10.1016/j.survophthal.2012.12.004 23642514

[B11] Gigante E. D. Taylor M. R. Ivanova A. A. Kahn R. A. Caspary T. (2020). ARL13B regulates sonic hedgehog signaling from outside primary cilia. Elife 9, e50434. 10.7554/eLife.50434 32129762 PMC7075693

[B12] Gotthardt K. Lokaj M. Koerner C. Falk N. Gießl A. Wittinghofer A. (2015). A G-protein activation Cascade from Arl13B to Arl3 and implications for ciliary targeting of lipidated proteins. Elife 4, e11859. 10.7554/eLife.11859 26551564 PMC4868535

[B18] Govers B. M. Van Huet R. A. C. Roosing S. Keijser S. Los L. I. Den Hollander A. I. (2023). The genetics and disease mechanisms of rhegmatogenous retinal detachment. Prog. Retin. Eye Res. 97, 101158. 10.1016/j.preteyeres.2022.101158 36621380

[B13] Han S. J. Jung J. K. Im S. S. Lee S. R. Jang B. C. Park K. M. (2018). Deficiency of primary cilia in kidney epithelial cells induces epithelial to mesenchymal transition. Biochem. Biophys. Res. Commun. 496, 450–454. 10.1016/j.bbrc.2018.01.079 29337054

[B14] Higginbotham H. Eom T. Y. Mariani L. E. Bachleda A. Hirt J. Gukassyan V. (2012). Arl13b in primary cilia regulates the migration and placement of interneurons in the developing cerebral cortex. Dev. Cell 23, 925–938. 10.1016/j.devcel.2012.09.019 23153492 PMC3529475

[B15] Humbert M. C. Weihbrecht K. Searby C. C. Li Y. Pope R. M. Sheffield V. C. (2012). ARL13B, PDE6D, and CEP164 form a functional network for INPP5E ciliary targeting. Proc. Natl. Acad. Sci. U. S. A. 109, 19691–19696. 10.1073/pnas.1210916109 23150559 PMC3511769

[B16] Labour M. N. Riffault M. Christensen S. T. Hoey D. A. (2016). TGFβ1 - induced recruitment of human bone mesenchymal stem cells is mediated by the primary cilium in a SMAD3-dependent manner. Sci. Rep. 6, 35542. 10.1038/srep35542 27748449 PMC5066273

[B17] Larkins C. E. Aviles G. D. East M. P. Kahn R. A. Caspary T. (2011). Arl13b regulates ciliogenesis and the dynamic localization of shh signaling proteins. Mol. Biol. Cell 22, 4694–4703. 10.1091/mbc.E10-12-0994 21976698 PMC3226485

[B19] Li Y. Wei Q. Zhang Y. Ling K. Hu J. (2010). The small GTPases ARL-13 and ARL-3 coordinate intraflagellar transport and ciliogenesis. J. Cell Biol. 189, 1039–1051. 10.1083/jcb.200912001 20530210 PMC2886347

[B20] Li J. Qi Y. Li B. Liu Y. Yang K. Zhang Z. (2023). STIL/AURKA axis promotes cell proliferation by influencing primary cilia formation in bladder cancer. J. Transl. Med. 21, 281. 10.1186/s12967-023-04118-2 37101292 PMC10131372

[B21] Lumi X. Hawlina M. Glavač D. Facskó A. Moe M. C. Kaarniranta K. (2015). Ageing of the vitreous: from acute onset floaters and flashes to retinal detachment. Ageing Res. Rev. 21, 71–77. 10.1016/j.arr.2015.03.006 25841656

[B22] Ma X. Xie Y. Gong Y. Hu C. Qiu K. Yang Y. (2023). Silibinin prevents TGFβ-Induced EMT of RPE in proliferative vitreoretinopathy by inhibiting Stat3 and Smad3 phosphorylation. Invest Ophthalmol. Vis. Sci. 64, 47. 10.1167/iovs.64.13.47 37906058 PMC10619698

[B23] Machemer R. Michels R. Okun E. Schepens C. Schwartz A. (1983). The classification of retinal detachment with proliferative vitreoretinopathy. Ophthalmology 90, 121–125. 10.1016/s0161-6420(83)34588-7 6856248

[B24] Ni H. Chen M. Dong D. Zhou Y. Cao Y. Ge R. (2024). CYLD/HDAC6 signaling regulates the interplay between epithelial-mesenchymal transition and ciliary homeostasis during pulmonary fibrosis. Cell Death Dis. 15, 581. 10.1038/s41419-024-06972-4 39122680 PMC11316090

[B25] Pennock S. Haddock L. J. Eliott D. Mukai S. Kazlauskas A. (2014). Is neutralizing vitreal growth factors a viable strategy to prevent proliferative vitreoretinopathy? Prog. Retin Eye Res. 40, 16–34. 10.1016/j.preteyeres.2013.12.006 24412519

[B26] Revenkova E. Liu Q. Gusella G. L. Iomini C. (2018). The Joubert syndrome protein ARL13B binds tubulin to maintain uniform distribution of proteins along the ciliary membrane. J. Cell Sci. 131, jcs212324. 10.1242/jcs.212324 29592971 PMC5992585

[B27] Seixas C. Choi S. Y. Polgar N. Umberger N. L. East M. P. Zuo X. (2016). Arl13b and the exocyst interact synergistically in ciliogenesis. Mol. Biol. Cell 27, 308–320. 10.1091/mbc.E15-02-0061 26582389 PMC4713133

[B28] Stocks S. Z. Taylor S. M. Shiels I. A. (2001). Transforming growth factor-beta1 induces alpha-smooth muscle actin expression and fibronectin synthesis in cultured human retinal pigment epithelial cells. Clin. Exp. Ophthalmol. 29, 33–37. 10.1046/j.1442-9071.2001.00368.x 11272783

[B29] Tamiya S. Liu L. Kaplan H. J. (2010). Epithelial-mesenchymal transition and proliferation of retinal pigment epithelial cells initiated upon loss of cell-cell contact. Invest Ophthalmol. Vis. Sci. 51, 2755–2763. 10.1167/iovs.09-4725 20042656

[B30] Thapa N. Wen T. Cryns V. L. Anderson R. A. (2023). Regulation of cell adhesion and migration via microtubule cytoskeleton organization, cell polarity, and phosphoinositide signaling. Biomolecules 13, 1430. 10.3390/biom13101430 37892112 PMC10604632

[B31] Wang X. Song X. Gao J. Xu G. Yan X. Yang J. (2024). Hedgehog/Gli2 signaling triggers cell proliferation and metastasis via EMT and wnt/β-catenin pathways in oral squamous cell carcinoma. Heliyon 10, e36516. 10.1016/j.heliyon.2024.e36516 39253258 PMC11382060

[B32] Xu J. Lamouille S. Derynck R. (2009). TGF-beta-induced epithelial to mesenchymal transition. Cell Res. 19, 156–172. 10.1038/cr.2009.5 19153598 PMC4720263

[B33] Yu X. Li L. Ning A. Wang H. Guan C. Ma X. (2024). Primary cilia abnormalities participate in the occurrence of spontaneous abortion through TGF-β/SMAD2/3 signaling pathway. J. Cell Physiol. 239, e31292. 10.1002/jcp.31292 38704705

[B34] Zhang Q. Li Y. Zhang Y. Torres V. E. Harris P. C. Ling K. (2016). GTP-binding of ARL-3 is activated by ARL-13 as a GEF and stabilized by UNC-119. Sci. Rep. 6, 24534. 10.1038/srep24534 27102355 PMC4840320

