# Supplementary material for: Loss of a primary cilia protein ARL13B promotes TGFβ-1 induced EMT of RPE in proliferative vitreoretinopathy via increasing Smad3 expression
Source: Front Cell Dev Biol. 2025 Dec 4;13:1661658. doi: 10.3389/fcell.2025.1661658 (PMC12711727; doi:10.3389/fcell.2025.1661658)

Supplementary Figures

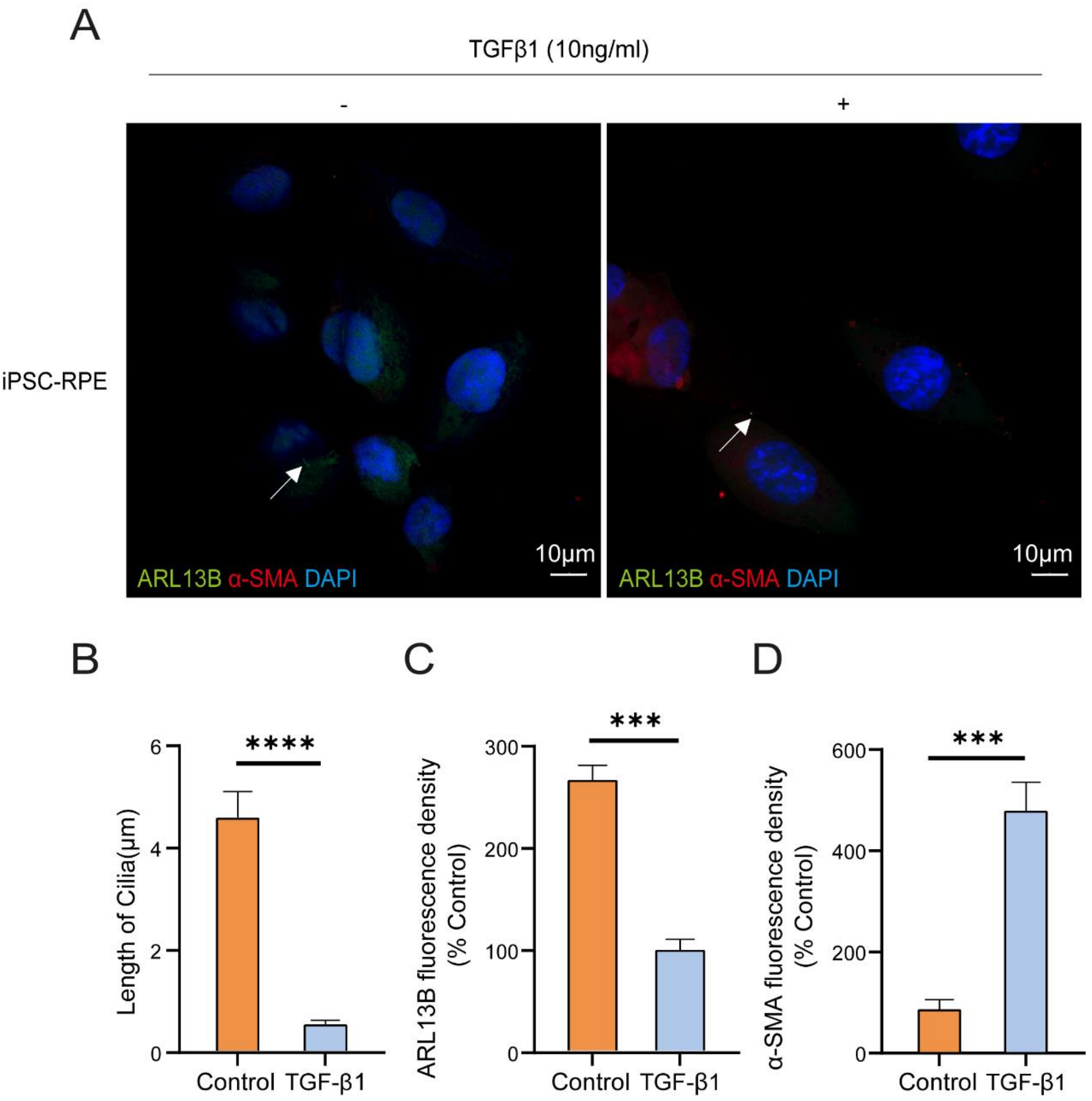

Figure S1 Reduced ARL13B protein levels in iPSC-RPE cells during EMT.

(A) Representative immunofluorescence staining in iPSC-RPE cells with TGF-β1-treated. Cilia (ARL13B, green), α-SMA (red), and nuclei (DAPI, blue). (B, C, D) Quantification of the cilia length (μm), ARL13B, and α-SMA fluorescence density in the control and TGF-β1-treated group. Data are represented as mean ± SD. n = 3, ***$p < 0.001$. **** $p < 0.0001$. Scale bar: 10 μm.

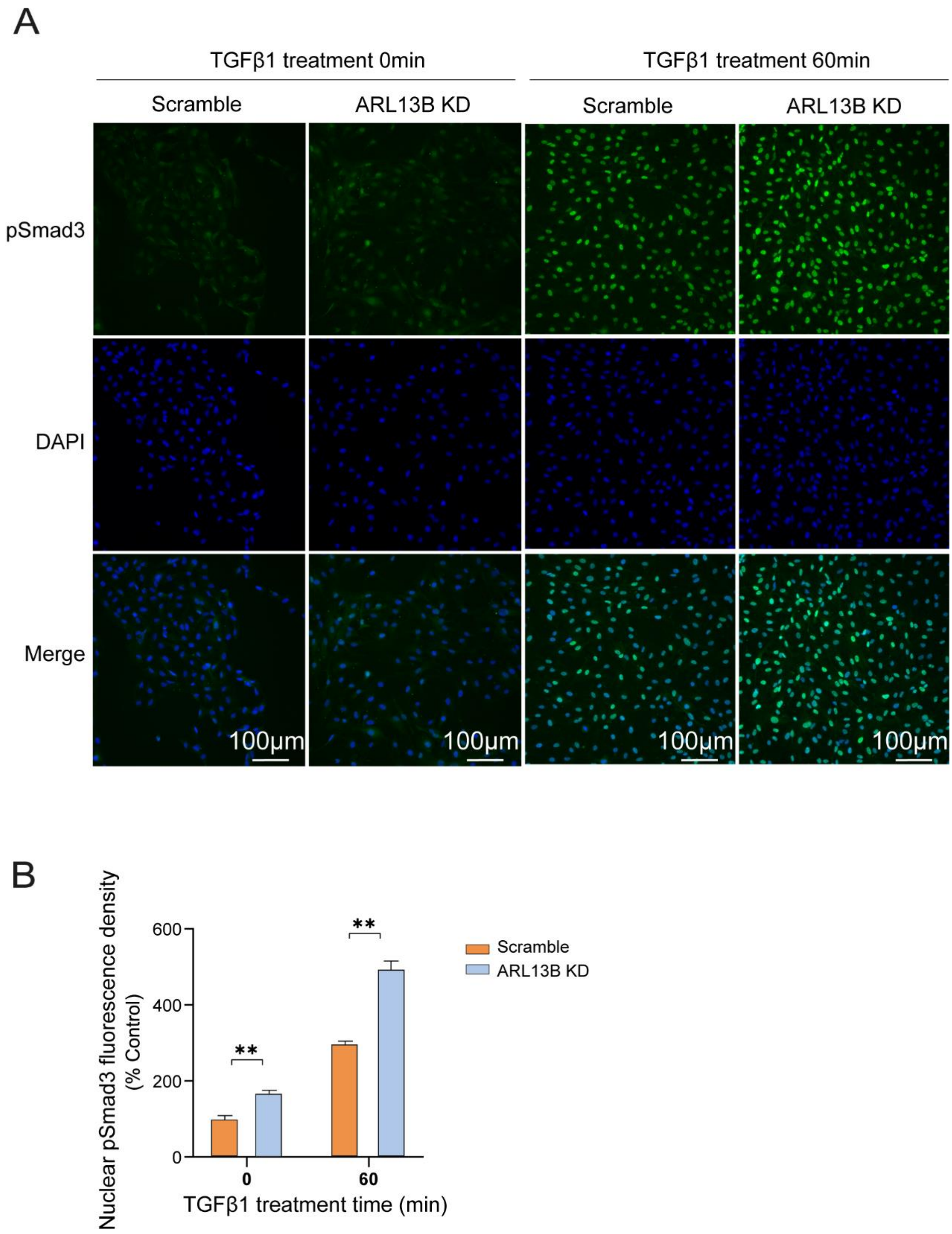

Figure S2 Nuclear translocation of pSmad3 in the RPE Cells with TGF-β1.

(A) Immunofluorescence staining of pSmad3 (green) in the scramble and ARL13B KD cells with TGF-β1 at 60 minutes, nuclei with DAPI (blue). (B) Quantification of the pSmad3 fluorescence density in (A). Data are represented as mean ± SD. ** $p < 0.01$.

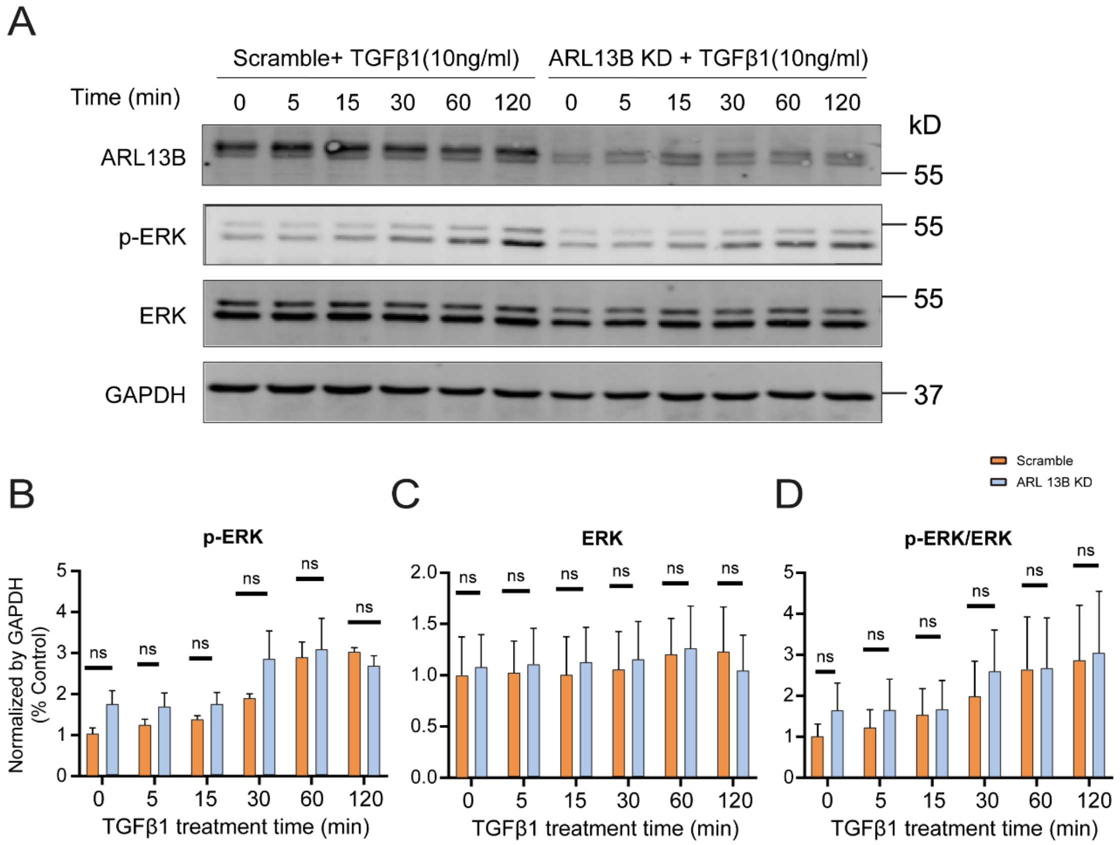

Figure S3 The effect of ARL13B on ERK pathway in RPE cells.

(A) Representative Western blots of ARL13B, p-ERK, and ERK in the scramble and ARL13B KD group with TGF-β1 at different times. (B; C; D) Quantification of indicated protein expression levels in (A), Data are represented as mean ± SD. ns $p > 0.05$.

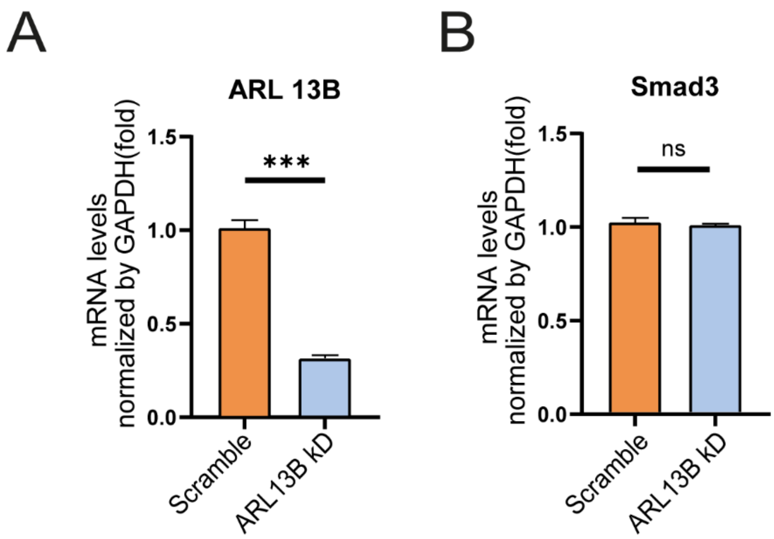

Figure S4 The effect of ARL13B on Smad3 in RPE cells.

(A; B) Quantification of indicated gene mRNA levels in the scramble and ARL13B KD group, ***P < 0.001; ns, no significance. Data are represented as mean ± SD. n = 3, ns $p > 0.05$, *** $p < 0.001$.

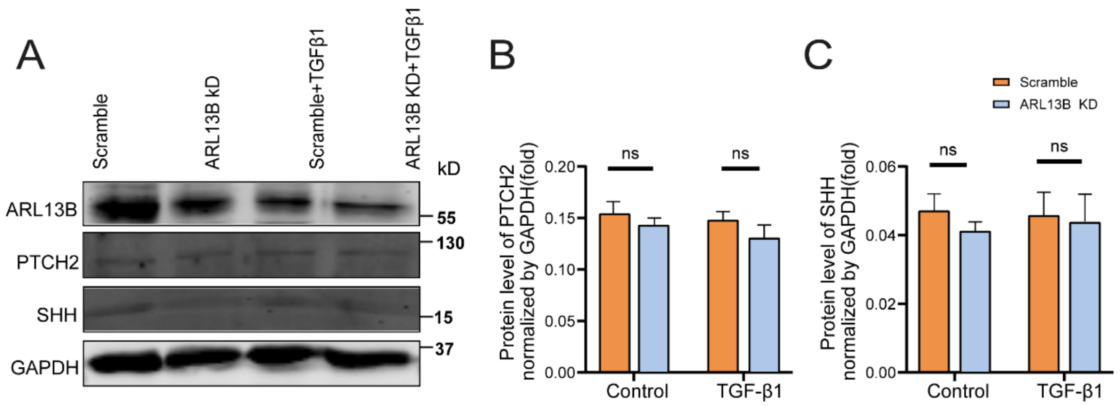

Figure S5 Effects of Hedgehog signaling on TGF-β1-induced EMT in RPE Cells.

(A) Representative Western blots of PTCH2 and SHH in scramble and ARL13B KD cells with/without TGF-β1. (B; C) Quantification of indicated protein expression levels in (A), Data are represented as mean ± SD. ns $p >$ 0.05.

Supplementary Table 1

	Supplementary Table 1. Primer used in Real-Time PCR
GAPDH	F-5’-CATGAGAAGTATGACAACAGCCT-3’
	R-5’-AGTCCTTCCACGATACCAAAGT-3’
ARL13B	F-5’-TGGTGGGACTTGATAATGCTGG-3’
	R-5’-GCTACATCTTCAGGGTATTCTCC-3’
Smad3	F-5’-TGACCACCAGATGAACCACAG-3’
	R-5’-CTGGCTGCAGGTCCAAGTT-3’

Fig 1D

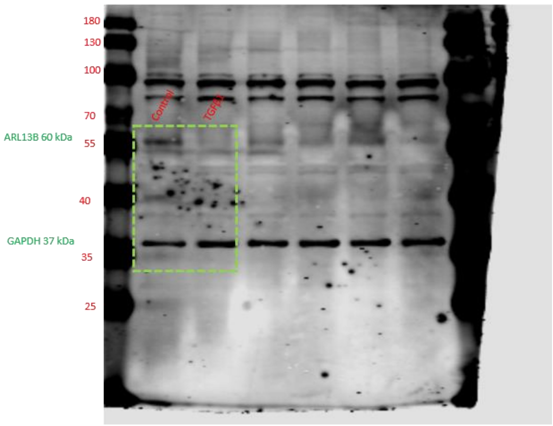

Fig 2A

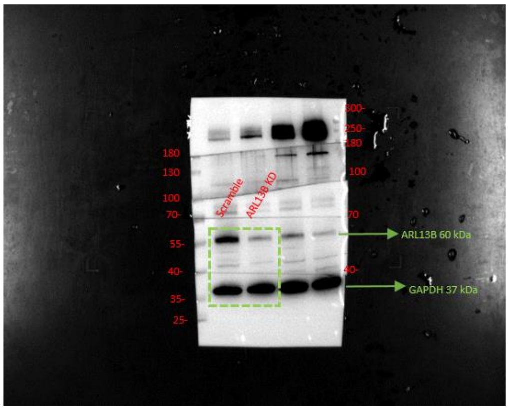

Fig 3B

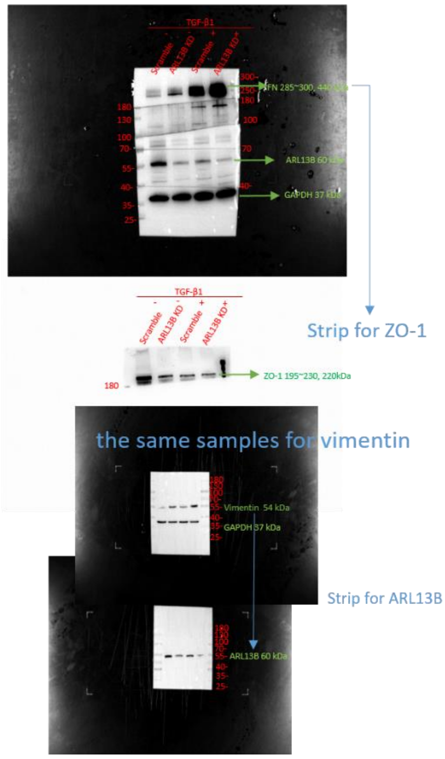

Fig 5C

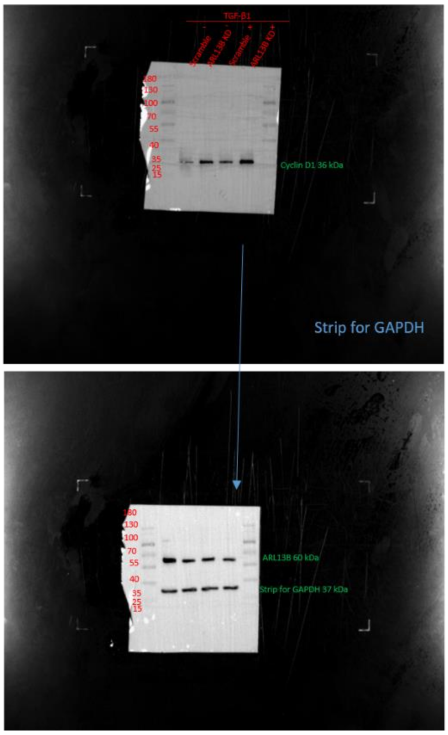

Fig 9B

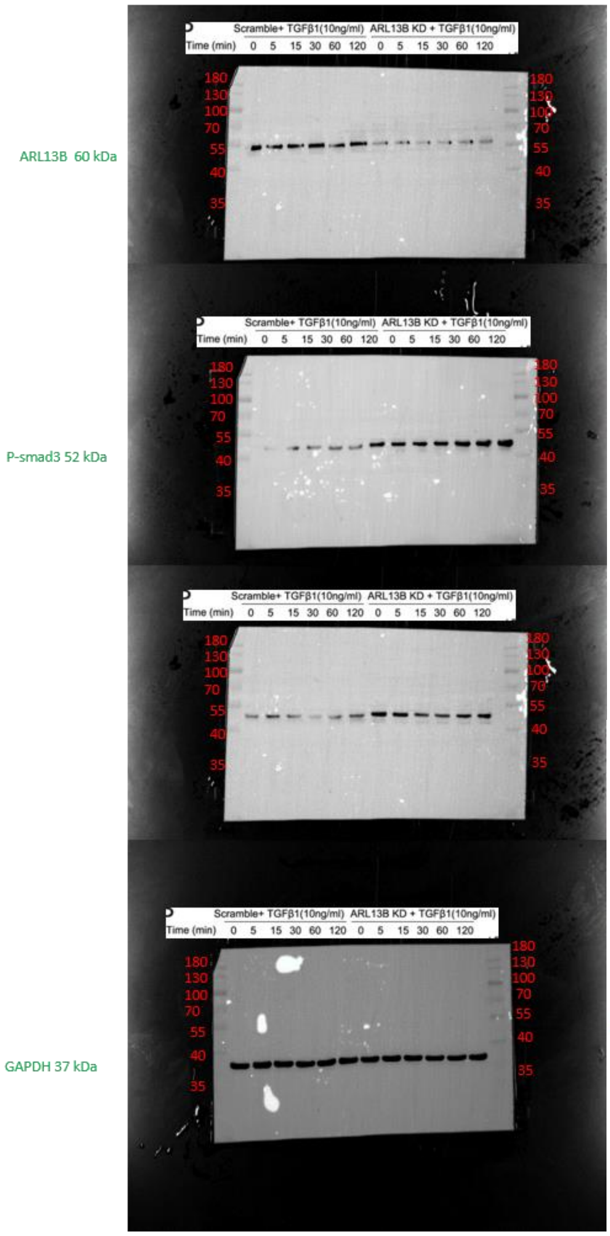

Fig S2 A

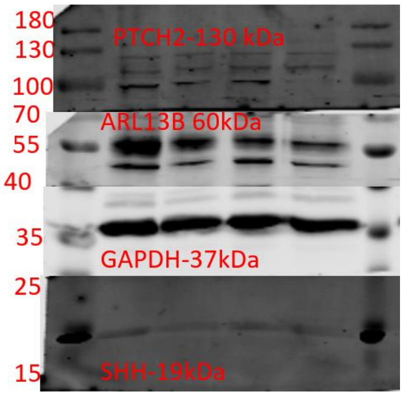

Fig S4 A

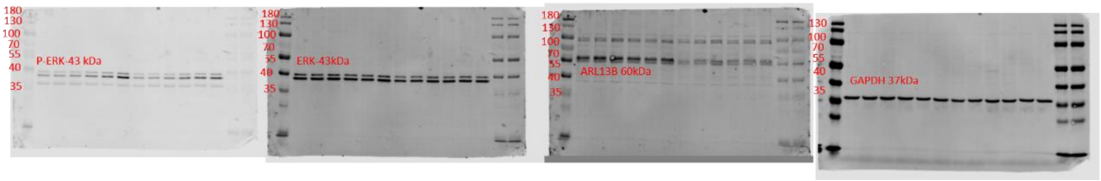

Supplement: Supplementary file 1 [file DataSheet1.pdf]
